# RelA Mutant *Enterococcus faecium* with Multiantibiotic Tolerance Arising in an Immunocompromised Host

**DOI:** 10.1128/mBio.02124-16

**Published:** 2017-01-03

**Authors:** Erin S. Honsa, Vaughn S. Cooper, Mohammed N. Mhaissen, Matthew Frank, Jessica Shaker, Amy Iverson, Jeffrey Rubnitz, Randall T. Hayden, Richard E. Lee, Charles O. Rock, Elaine I. Tuomanen, Joshua Wolf, Jason W. Rosch

**Affiliations:** a Department of Infectious Diseases, St. Jude Children’s Hospital, Memphis, Tennessee, USA; b Department of Microbiology and Molecular Genetics, University of Pittsburgh, Pittsburgh, Pennsylvania, USA; c Department of Oncology, St. Jude Children’s Hospital, Memphis, Tennessee, USA; d Department of Pathology, St. Jude Children’s Hospital, Memphis, Tennessee, USA; e Department of Chemical Biology and Therapeutics, St. Jude Children’s Hospital, Memphis, Tennessee, USA; f Department of Pediatrics, University of Tennessee Health Science Center, Memphis, Tennessee, USA; Harvard Medical School

## Abstract

Serious bacterial infections in immunocompromised patients require highly effective antibacterial therapy for cure, and thus, this setting may reveal novel mechanisms by which bacteria circumvent antibiotics in the absence of immune pressure. Here, an infant with leukemia developed vancomycin-resistant *Enterococcus faecium* (VRE) bacteremia that persisted for 26 days despite appropriate antibiotic therapy. Sequencing of 22 consecutive VRE isolates identified the emergence of a single missense mutation (L152F) in *relA*, which constitutively activated the stringent response, resulting in elevated baseline levels of the alarmone guanosine tetraphosphate (ppGpp). Although the mutant remained susceptible to both linezolid and daptomycin in clinical MIC testing and during planktonic growth, it demonstrated tolerance to high doses of both antibiotics when growing in a biofilm. This biofilm-specific gain in resistance was reflected in the broad shift in transcript levels caused by the mutation. Only an experimental biofilm-targeting ClpP-activating antibiotic was able to kill the mutant strain in an established biofilm. The *relA* mutation was associated with a fitness trade-off, forming smaller and less-well-populated biofilms on biological surfaces. We conclude that clinically relevant *relA* mutations can emerge during prolonged VRE infection, causing baseline activation of the stringent response, subsequent antibiotic tolerance, and delayed eradication in an immunocompromised state.

## IMPORTANCE

The increasing prevalence of antibiotic-resistant bacterial pathogens is a major challenge currently facing the medical community. Such pathogens are of particular importance in immunocompromised patients as these individuals may favor emergence of novel resistance determinants due to lack of innate immune defenses and intensive antibiotic exposure. During the course of chemotherapy, a patient developed prolonged bacteremia with vancomycin-resistant *Enterococcus faecium* that failed to clear despite multiple front-line antibiotics. The consecutive bloodstream isolates were sequenced, and a single missense mutation identified in the *relA* gene, the mediator of the stringent response. Strains harboring the mutation had elevated baseline levels of the alarmone and displayed heightened resistance to the bactericidal activity of multiple antibiotics, particularly in a biofilm. Using a new class of compounds that modulate ClpP activity, the biofilms were successfully eradicated. These data represent the first clinical emergence of mutations in the stringent response in vancomycin-resistant entereococci.

## INTRODUCTION

Current challenges in treatment of infections are focused on the marked reduction of new candidates in the antibacterial discovery pipeline at a time of increasing rates of antimicrobial resistance, including emergence of methicillin-resistant *Staphylococcus aureus* (MRSA), β-lactam- and macrolide-resistant *Streptococcus pneumoniae*, and vancomycin-resistant enterococci (VRE) (1–4). VRE species are especially problematic clinically, as these bacteria are resistant to all first-line antibiotics and infection is associated with a marked increase in risk of mortality (5–8). However, other challenges to therapeutic success are also emerging, particularly in the immuncompromised host, where refractory bacteremia and prolonged antibiotic therapy increase the opportunity to select for alternative bacterial survival traits by mutation or genetic exchange (9–12). Once mutations arise, the permissive nature of the compromised immune system may allow for the development of secondary mutations that compensate for any associated fitness trade-offs (13). As such, hosts more permissive for infection have been postulated to represent an important reservoir for the emergence of novel problematic pathogens (14).

In the absence of host defenses, bacterial killing by antibiotics is required for cure. Commonly this is ensured by administering drugs with the aim of ensuring that concentrations at the site of infection are above the MIC as reported by the clinical laboratory. This assumes the bactericidal concentration is close to the MIC. However, bacteria can persist when the MIC and minimal bactericidal concentration (MBC) dissociate such that antibiotics inhibit growth but fail to kill, a property called “tolerance” (15–17). As genome sequencing technologies improve, it has become evident that mutations—particularly those involved in stress responses—can impact efficacy of antibiotics (18). The bacterial stringent response, which slows metabolism under low-nutrient or stress conditions, can decrease the response to antibiotic therapy, allowing quiescent bacteria to survive and tolerate antibiotics, without a change in MIC (19). In *S. aureus*, induction of the stringent response increases tolerance to β-lactam antibiotics (20–22), and in Gram-negative bacteria, activation contributes to the production of biofilm persister cells (23, 24). Also, a report on *Enterococcus faecalis* demonstrated that treatment of cells with mupirocin induced expression of transport and stress-related genes, and strong repression of genes involved DNA, RNA, and protein synthesis, similar to a stringent response (25). Despite the importance of the stringent response, there are few reports of clinical emergence of mutations in this pathway, none demonstrating reduced antibiotic efficacy (22), and no studies focusing on antibiotic tolerance and stringent response in *E. faecium*. For *Enterococcus* species, although the stringent response pathway is well characterized and antibiotic resistance is widespread, we report the first example of mutation in the stringent response pathway causing increased baseline alarmone levels, which was responsible for antibiotic tolerance within a biofilm.

## RESULTS

### Clinical setting.

The patient was a 6-week-old African-American girl born by normal vaginal delivery at term. Acute myeloid leukemia was diagnosed at 4 weeks of age, after she presented with fever and marked leukocytosis. A double-lumen central venous catheter (CVC) was placed (7.0 French-scale Hickman catheter; Bard Access Systems, Salt Lake City, UT), and chemotherapy was initiated, resulting in prolonged profound neutropenia. Induction chemotherapy comprised systemic cytarabine, daunorubicin, etoposide, and methylprednisolone plus intrathecal methotrexate, hydrocortisone, and cytarabine. The patient received intravenous cefepime as antibacterial prophylaxis. After 2 weeks of chemotherapy, during profound neutropenia, routine microscopic examination of a peripheral blood smear revealed bacterial organisms, although the patient had no signs or symptoms of infection. Blood cultures were obtained from both lumens of the CVC, and empirical combination therapy with vancomycin and meropenem was initiated. After blood cultures grew vancomycin-resistant *Enterococcus faecium*, the antibiotic regimen was changed to linezolid, which was eventually supplemented with daptomycin, gentamicin, and quinupristin-dalfopristin (Table 1).

**TABLE 1:**
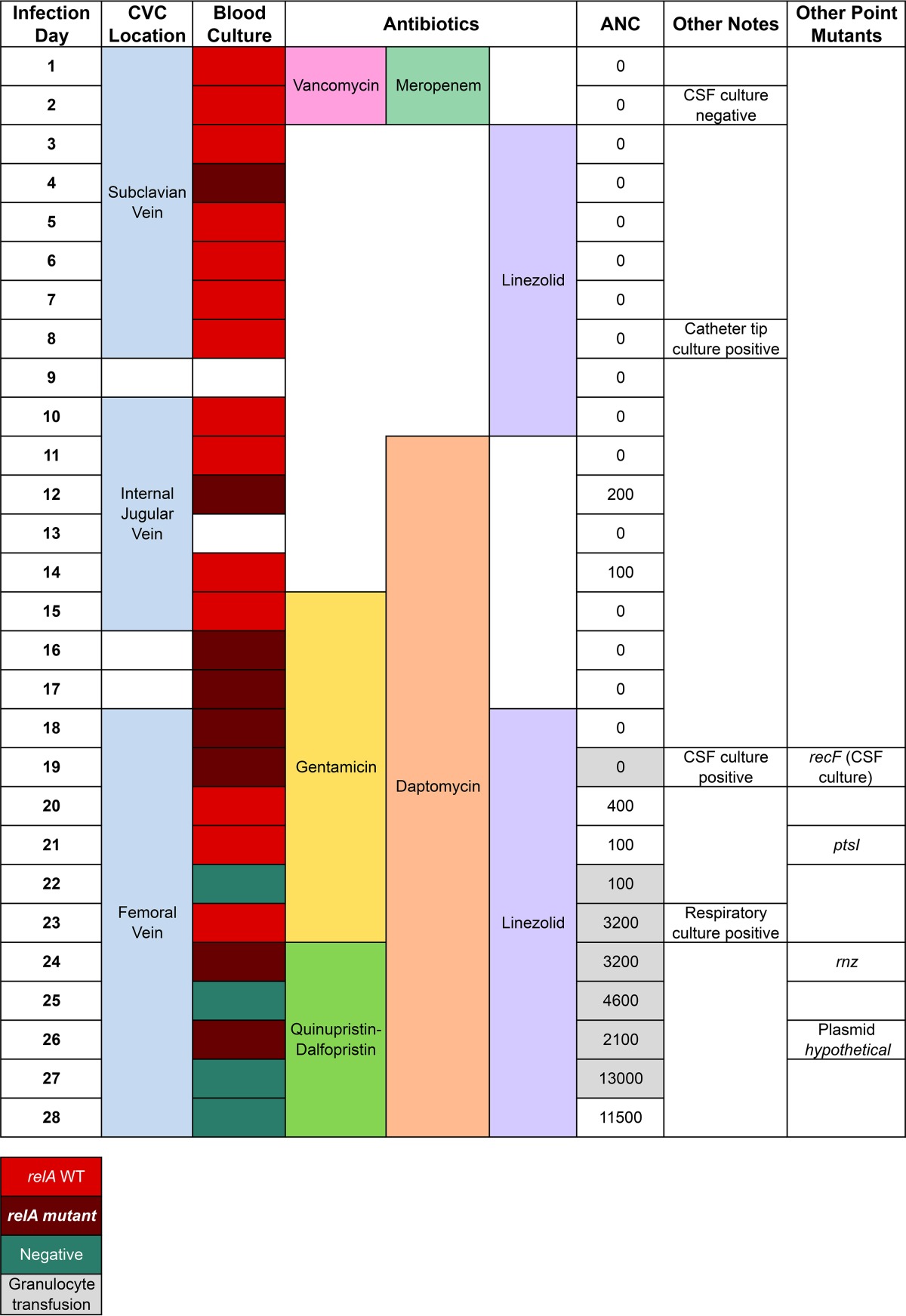
Summary of patient parameters and antimicrobial therapy^a^

a ANC, absolute neutrophil count; CVC, central venous catheter; CSF, cerebrospinal fluid.

Initial paired blood cultures drawn from the two CVC lumens showed a differential time to positivity of 4.0 h, and culture of the explanted device at the time of CVC removal (day 9) was positive for *E. faecium*, suggesting that the infection was initially related to biofilm on the surface of the CVC (26). However, over the 28 days, the bacteremia failed to clear despite targeted antibiotic therapy, as well as CVC removal and replacement on two occasions, both allowing for CVC-free periods. Antibiotic therapy was chosen in accordance with expert opinion and *in vitro* susceptibility testing that indicated the strain remained sensitive to all antibiotics administered except the initial vancomycin and meropenem (27). During the persistent bacteremia, there was no significant change in the reported antimicrobial susceptibility pattern according to testing performed in our clinical microbiology laboratory. Every strain was susceptible to linezolid (MIC, 1 to 2 µg/ml), quinupristin-dalfopristin (MIC, 0.5 µg/ml), and daptomycin (MIC, 3 to 4 µg/ml). High-level resistance to gentamicin was not detected (MIC, <500 µg/ml).

A comprehensive diagnostic evaluation, including Doppler ultrasonography of the upper and lower extremities to detect intravascular thrombosis, ultrasound of head and abdomen, and magnetic resonance imaging (MRI) of the entire body failed, to identify any additional source of persistent infection. Analysis of cerebrospinal fluid (CSF) obtained through lumbar puncture on day 2 of infection was within normal limits, and bacterial culture was sterile. Transthoracic echocardiography performed on days 11 and 22 showed mild preexisting aortic regurgitation without clear evidence of vegetations. The initial source for the bloodstream infection is thought to have been luminal colonization and biofilm formation within the CVC, and after device removal, the bloodstream infection persisted. Based on this history, we believe that the most likely focus of persistent infection was occult endocarditis or septic thrombophlebitis.

The patient’s absolute neutrophil count (ANC) remained at 0 cells/mm^3^ until day 20, when donor granulocyte transfusion was initiated. Granulocyte transfusion was repeated again on days 22, 23, 24, 25, 26, and 27 until neutropenia resolved. Daily ANC values are shown in Table 1. On day 27, the bacteremia cleared, and the patient received an additional 6 weeks of linezolid treatment for possible endocarditis with full clinical recovery.

### Genetic characterization.

The initial VRE isolate was sequenced and completely assembled to obtain a closed genome comprising one 2.93-Mbp chromosome and three plasmids of 171, 78.6, and 59.2 kbp, containing a total of approximately 3,208 open reading frames (see Table S1 in the supplemental material). Analysis of the functional roles of predicted coding regions revealed multiple antibiotic resistance systems, including the *vanB* gene cluster for vancomycin, fluoroquinolones, aminoglycosides, β-lactams, and multiple putative drug efflux pumps (Table 2) (28, 29). All 22 isolates were completely sequenced at high depth (mean coverage of 256.6×) and aligned to the closed reference genome of the initial VRE isolate. In total, all genomes differed by only five single nucleotide polymorphisms (SNPs), and no insertions or deletions were detected, indicating that a single strain was responsible for the prolonged bacteremia. Four of the five mutations were identified only once in single isolates: therefore, we concluded these mutations were not clinically relevant. These mutations included Y175C missense mutation in *rnz* (RNase Z), the A221D missense mutation in *ptsI* (phosphoenolpyruvate phosphotransferase enzyme I), the P640H missense mutation in *recF* (recombination protein F), and a silent mutation in a hypothetical plasmid protein (Table 1). However, one missense mutation in the *relA* gene, predicted to encode an RelA(L152F) variant, was found in eight isolates. The *relA* mutation was first detected 3 days after starting antibiotic therapy and persisted through the clinical course until the infection eventually resolved (Table 1). The observation that the mutation was intermittently identified indicated that both the initial strain and the* relA* mutant strain coexisted until the later stages of the infection. As will be discussed, there may have been initial sampling bias during collection of a representative isolate from each day listed in Table 1; however, we believe this may have underestimated the true presence and persistence of the *relA* variant subpopulation.

10.1128/mBio.02124-16.3 Table S1  Genomic characterization of the VRE isolate. Download Table S1, XLS file, 2.1 MB. Copyright © 2017 Honsa et al. 2017 Honsa et al. This content is distributed under the terms of the Creative Commons Attribution 4.0 International license.

**TABLE 2:** VRE isolates possessed multiple antibiotic resistance genes

Resistance function	Antibiotic	Resistance gene(s)
Alter cell wall charge	Polymyxin	*pmrE*
Antibiotic-altering enzyme	Macrolide	*ermB*, *msrC*
Antibiotic efflux	Fluoroquinolone	*arlR*
Antibiotic efflux	Tetracycline	*adeC*,* tetK*,* tetC*
Antibiotic efflux	Wide range	*lmrCD*
Antibiotic efflux	Lincosamide	*lsaE*
Antibiotic efflux	Streptogramin	*msrC*, *isaA*
Antibiotic inactivation enzyme	Lincosamide	*lnuB*
Aminoglycoside-modifying enzymes	Aminoglycoside	*aac(6′)-Ii*, aac*(6′)-Ie*–*aph(2′′)-Ia*
Aminoglycoside-modifying enzymes	Aminoglycoside	*aph(3′)-IIIa*,* aad(6′)*
Antibiotic target protection protein	Tetracycline	*tet32*,* tetO*
Antibiotic target replacement proteins	β-Lactams	*pbp1b*,* pbp2x*,* pbp2b*, *pbp1a*, *mecC*
Molecular bypass	Glycopeptide	*vanB* cassette
Target Mutation	Trimethoprim	*dfrE*, *dfrF*
Target mutation	Rifampin	*rpoB*
Target mutation	Fluoroquinolone	*gyrB*

RelA is a critical mediator of the bacterial stringent response via production of the alarmone guanosine tetraphosphate (ppGpp) and has been implicated in resistance or tolerance to antibiotic stress in several bacterial pathogens (30). In our RelA mutants, the altered residue (L152F) is immediately adjacent to residues essential for the hydrolase activity of the RelA enzyme (31). Modeling of the structural consequences of this mutation indicated that the native leucine is buried in the structure; however, replacement of this residue with a phenylalanine would not allow for such tight packing, potentially causing a local change in the active site. This mutation was initially detected on the fourth day of the bloodstream infection and appeared to become more prevalent later in the course of therapy, particularly once daptomycin was included in the antibiotic regimen (Table 1). Previous studies have also identified mutations in *relA* in response to daptomycin exposure *in vitro*, although no impact on antibiotic efficacy has been demonstrated (32). These data indicate that the VRE population remained mostly homogeneous, except for a single-point mutation in the stringent response pathway. This *relA* mutation arose during the course of the infection and persisted throughout the course of therapy despite treatment with antibiotics to which the bacterium retained apparent susceptibility based on MIC testing. It is worth noting that the VRE strains analyzed in this paper were genetically identical (isogenic), except for the *relA* missense mutation, and they all possessed multiple antibiotic resistance genes as shown in Table 2. Furthermore, no growth defects were identified between the wild type (WT) and the isogenic *relA* mutants in the absence of stressors (see Fig. S1A in the supplemental material).

10.1128/mBio.02124-16.1 Figure S1  No planktonic growth defects were detected in either the WT or isogenic *relA* mutant. Download Figure S1, DOCX file, 0.1 MB. Copyright © 2017 Honsa et al. 2017 Honsa et al. This content is distributed under the terms of the Creative Commons Attribution 4.0 International license.

### Effects of RelA mutation.

**FIG 1:**
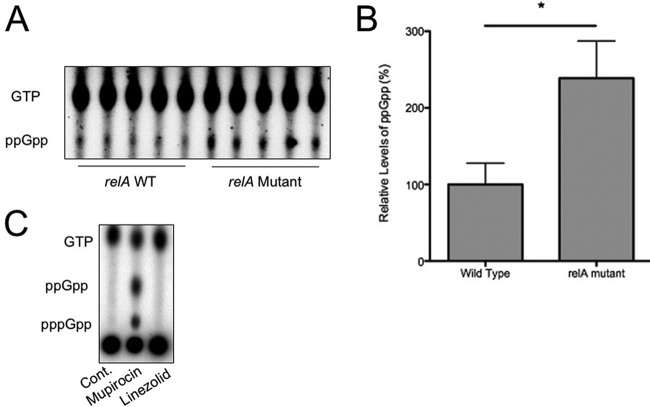
Basal levels of ppGpp are increased in VRE strains harboring mutant *relA* alleles. (A) Multiple *relA* WT or mutant (isogenic) strains were grown in low-phosphate media, followed by incubation with ^32^P, and levels of resting (p)ppGpp were measured by densitometry. (B) Data for ppGpp levels are quantified, and means and standard deviations are shown. Levels were normalized relative to the wild-type strain (100%). *, *P* = 0.0079 by Mann-Whitney testing. (C) Controls: negative control without any stressor (Cont.), positive-control mupirocin, as well as linezolid.

The missense mutation in a highly conserved domain essential for RelA hydrolase activity led us to ascertain whether the identified mutation in *relA* conferred any discernible differences in levels of (p)ppGpp, the alarmone that mediates the downstream stringent response pathways. Strains that were genetically identical with the exception of the single-base-pair *relA* mutation were allowed to incorporate ^32^P, and the baseline levels of (p)ppGpp were measured. Strains carrying the mutation in *relA* demonstrated approximately 3× greater basal levels of ppGpp than the WT controls (Fig. 1A, thin-layer chromatography [TLC] blot, and B, quantification [*P* < 0.001, Mann-Whitney test]). This observation suggested inappropriate activation of the alarmone at baseline in the absence of an external stimulus, potentially priming the cells to more rapidly adapt to adverse conditions such as antibiotic exposure. While we attempted to monitor differences in (p)ppGpp and GTP levels in linezolid-stressed *relA* WT and mutant strains, we could not detect any induction of the stringent response (Fig. 1C). It should be noted that both WT and *relA* mutant bacteria stressed with mupirocin did not show a difference in activated stringent response alarmone levels (WT shown in Fig. 1C [mutant identical to WT]). However, as will be discussed, we suggest the fitness benefit of the *relA* mutation is to provide a higher resting level of alarmone, which allowed these strains to better adapt and respond to antibiotic stress during therapy (tolerance).

**FIG 2:**
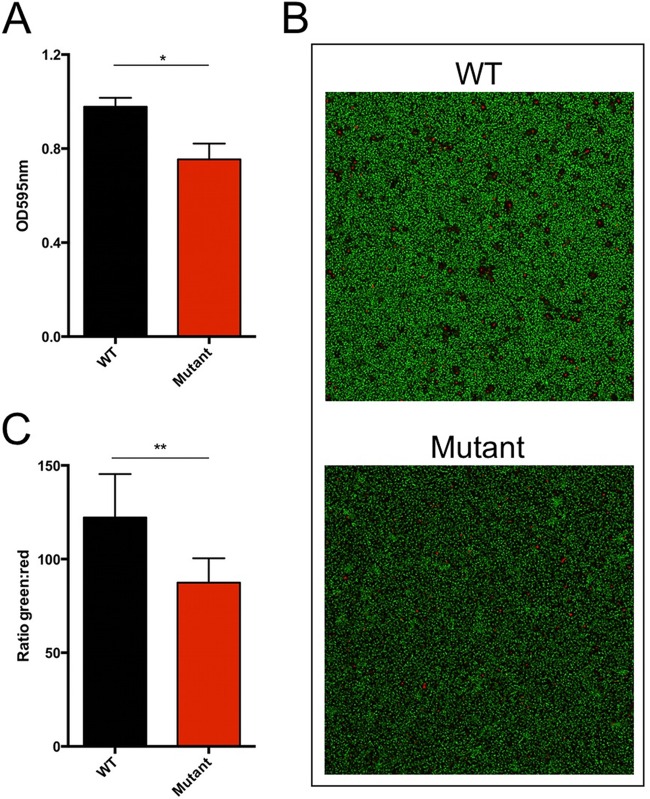
Biofilm size and live cell number are decreased in VRE strains harboring mutant *relA* alleles. (A) Biofilms of the VRE WT and *relA* mutant were formed in 24-well plates coated with bovine fibronectin. Quantification of biofilm was performed with crystal violet staining. Absorbance at 595 nm was measured for each sample. Three independent experiments were performed and combined to determine the mean average (with error bars). *, *P* = 0.0074 by two-tailed *t* test. The *relA* WT consistently grew to 10^9^ CFU/ml, while the *relA* mutant grew to 10^8^ CFU/ml per 24-h biofilm. (B and C) Biofilms were formed on multichambered glass slides. After 48 h, biofilms were stained with the Live/Dead BacLight bacterial viability kit and analyzed by confocal microscopy. (B) Representative images of a single segment. (C) The ratio of green to red (fluorescein isothiocyanate [FITC] to tetramethyl rhodamine isothiocyanate [TRITC]) signal was calculated from the average of each section, and ratios were combined for the final average (*n* = 8). **, *P* = 0.002.

The most likely initial focus of infection in this case, the CVC, is associated with the development of surface-associated bacterial biofilm (33–35). This was possibly followed by biofilm formation on the heart valves; however, this could not be conclusively diagnosed. As mutations in* relA* have been implicated in alterations in biofilm formation, we next determined the effect of this mutation on the capacity of these strains to form biofilm. Since traditional growth curves (see Fig. S1B in the supplemental material) and time-kill assays (see Fig. S2 in the supplemental material) did not detect differences in the survival of the *relA* mutant compared to an isogenic *relA* WT strain (planktonic growth) in the presence of daptomycin or linezolid, we hypothesized that the mutation may demonstrate a more pronounced phenotype in a biofilm population. To explore a possible fitness trade-off in biofilm formation, we grew biofilms on fibronectin-coated 24-well plates for 24 h. After removal of any planktonic cells and staining with crystal violet, absorbance at 595 nm was determined as a quantification of biofilm size and thickness. Parallel to this analysis, we resuspended non-crystal violet-treated biofilms in phosphate-buffered saline (PBS) and serially diluted and plated for CFU-per-milliliter counts. As shown in Fig. 2A, the *relA* mutant consistently had significantly smaller biofilms than the isogenic WT strain when grown on these fibronectin-coated surfaces. Concurrently, identically grown biofilms identified at least a log decrease of *relA* mutant biofilm growth compared to the WT isogenic strain (10^9^ versus 10^8^ CFU after 24 h of growth in fibronectin-coated plates). Each strain was grown under identical conditions, as well as inoculated from titrated stocks, indicating that the missense mutation in *relA* conferred a defect in biofilm formation and bacterial survival within a biofilm.

10.1128/mBio.02124-16.2 Figure S2  Time-kill assays of the WT and *relA* mutant. Download Figure S2, DOCX file, 0.1 MB. Copyright © 2017 Honsa et al. 2017 Honsa et al. This content is distributed under the terms of the Creative Commons Attribution 4.0 International license.

To further investigate defects in biofilm formation of our *relA* mutant, we performed confocal fluorescence microscopy. Here, biofilms of *relA* WT and mutant isogenic strains were allowed to form on an abiotic surface (microscope slide chamber) for 48 h. Live/dead staining was then performed, and confocal microscopy was used to measure fluorescein isothiocyanate (FITC) and tetramethyl rhodamine isothiocyanate (TRITC) fluorescence of z-stack 16-µm sections of each biofilm. To remove bias from biofilm sampling, each chamber for each strain was randomly analyzed in four separate sections, and each strain was analyzed in two separate chambers on multiple days, allowing an *n* value of 8 for each strain. Figure 2B shows that the WT strain had consistently brighter fluorescence in each section, compared to the *relA* mutant on the right. Furthermore, when the green and red signals were quantified and a ratio determined (Fig. 2C), there was a consistently higher fluorescent intensity for live cells for the WT strains. This suggests that the biofilm of the mutant harbors less total live cells, which correlates back to the CFU/ml data of established cells. Together, our biofilm analysis suggests that the ability to form thicker, more robust biofilms is compromised in the *relA* mutant.

A mutation such as *relA*(L152F) that primes cells to enter the stringent response by increasing ppGpp levels is expected to affect expression of many genes. Likewise, the phenotypic differences between planktonic and biofilm growth of these strains should be related to transcriptome changes. We tested these predictions by performing RNA sequencing on these strains grown either in the liquid planktonic phase or as biofilms on a biotic surface. This experiment revealed broad changes in expression in as much as 15% of the ~2,940 genes in the genome in the *relA* mutant compared to the WT and during the shift from planktonic to biofilm growth. The interactions between genotype and growth environment were most telling. Not only did this single *relA* mutation affect expression (mostly by downregulation) of a broad array of genes in both conditions, it also changed how certain genes responded during the shift from planktonic to biofilm growth. In fact, 35 genes responded in opposite directions in the shift to biofilm growth for the WT and the mutant (i.e., expression was up for the WT, down for the mutant, or vice versa), and 25 genes were divergently expressed for the *relA* mutant under biofilm and planktonic growth conditions. Some of these genes with divergent expression provide potential mechanistic explanations for the biofilm-dependent resistance observed, including *liaS*, a major target of mutations causing resistance to daptomycin (36), which was upregulated in the WT biofilm relative to WT planktonic culture, but downregulated in the *relA* biofilm relative to planktonic *relA* cells. A small group of genes were uniquely upregulated in the *relA* biofilm, including genes with chaperone functions, such as *greA* (EFAU004_01358) and *cspA* (EFAU004_01491), which would presumably enhance stress tolerance. However, the overwhelming signature of the *relA* mutant was that it downregulated far more genes during biofilm growth than the WT strain (480 genes in comparison with 214 genes), and the direct comparison of the WT biofilm and the *relA* biofilm also revealed 433 genes downregulated by the mutant. In summary, this single mutation not only suppressed expression of many genes, it did so in a biofilm-dependent manner that actually reversed the tendency of some genes to be upregulated under this condition by the WT.

**FIG 3:**
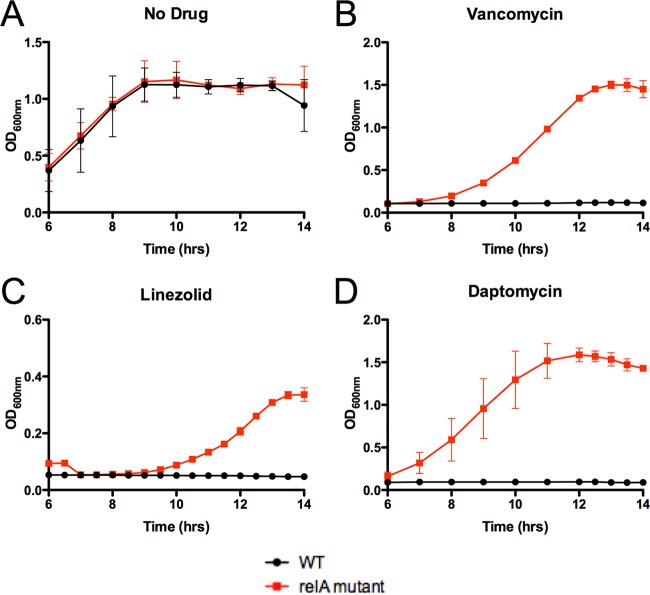
The *relA* mutation contributes to tolerance to antibiotics in biofilms. Strains were grown and allowed to form biofilms by using a peg suspension system. Biofilms were then treated with the respective antibiotics (256 µg/ml vancomycin, 50 µg/ml linezolid, and 50 µg/ml daptomycin) for 24 h. After antibiotic treatment, biofilms were washed, collected by centrifugation, and allowed to outgrow in antibiotic-free medium to ascertain an approximation of bacterial killing. Data represent the mean of three replicates; error bars represent standard deviation. For clarity, 6 h to 14 h is shown, as there was no growth prior to 6 h for drug-treated VRE.

Given these biofilm-dependent changes in expression, it was somewhat surprising to observe a fitness trade-off for the *relA* mutant in forming biofilms in standard media. However, in the patient, the bacteria would have been constantly exposed to various antibiotic stresses during therapy. We therefore investigated whether the *relA* mutation conferred enhanced tolerance to antibiotic-mediated killing in a biofilm model. Since no resistance was detected against linezolid and daptomycin (MIC clinical data and Table 2), we hypothesized that the *relA* mutation was not responsible for a detectable rise in antibiotic resistance, but rather allowed the mutants to respond faster to antibiotic stress in a biofilm, thus becoming tolerant to multiple antibiotics. Biofilms of the two isogenic strains (WT or *relA* mutant) were allowed to form on Nunc peg plates in the rich medium Todd-Hewitt broth supplemented with yeast extract, following previously published protocols (37, 38). As a control, the *relA* mutation conferred no discernible difference in the outgrowth of planktonic cells removed from biofilms in the absence of antibiotics (Fig. 3A), indicating the *relA* mutation did not affect outgrowth from a biofilm. However, when the biofilms were subjected to prolonged antibiotic exposure, high concentrations of vancomycin, linezolid, and daptomycin were successful in eradicating the WT strain but failed to eradicate strains harboring the *relA* mutation (Fig. 3B, C, and D). It should be noted that the subsequent outgrowth of each strain was in the absence of antibiotic, so no antibiotic carryover could be responsible for the difference in response to antibiotic stress. These data indicated that this mutation conferred a selective advantage to the *in vitro* survival of antibiotic-tolerant persister cells within a biofilm, in the absence of linezolid or daptomycin resistance using standard CLSI susceptibility testing. Despite the defect of biofilm formation on fibronectin-coated plates, the *relA* mutant still displayed enhanced tolerance to antibiotics in the biofilm state. Taken together, these data suggest that while the *relA* mutation led to increased alarmone resting levels and subsequent tolerance to multiple-antibiotic stressors, the mutant strain was deficient in the ability to form biofilms compared to its WT counterparts.

**FIG 4:**
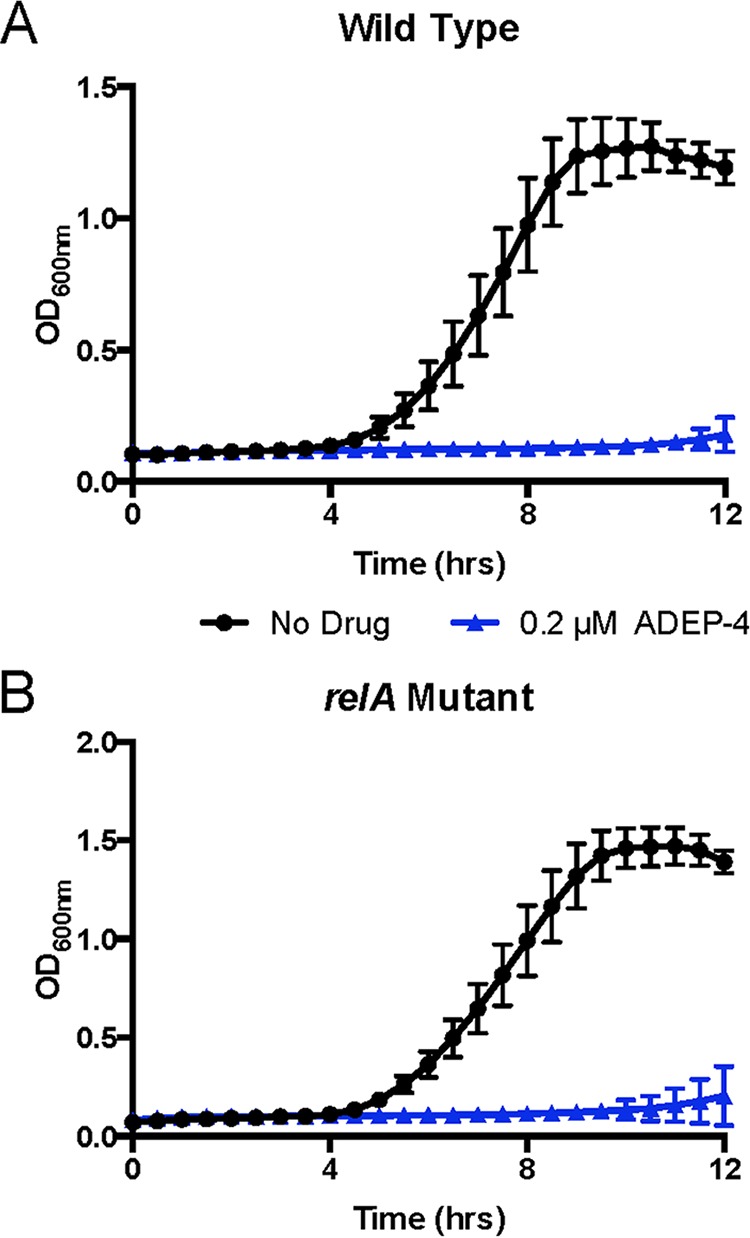
The ClpP activator successfully eradicates biofilm irrespective of the *relA* mutation. The wild type (A) and an isogenic mutant (B) harboring the *relA* missense mutation were allowed to form biofilms for 24 h. Following biofilm formation, cells were treated with either DMSO (black lines) or the ClpP activator ADEP-4 at 0.2 µM for 24 h (blue lines). After antibiotic treatment, biofilms were washed, collected by centrifugation, and allowed to outgrow in antibiotic-free medium to ascertain an approximation of bacterial killing. Data represent the mean from four replicates; error bars represent standard deviation.

Eradication of bacterial biofilms is a major challenge during therapy due to their inherently greater tolerance to many antibiotics and their capacity to both resist antibiotic penetration and resist immune clearance (39). This phenomenon is especially confounded in immunocompromised patients. A new experimental approach to target such refractory bacterial communities has been the development of a class of compounds that cause unregulated activation of the housekeeping protease ClpP (40). We assayed the bactericidal activity of one such investigational compound, ADEP-4, against isogenic strains possessing either WT or *relA* mutant strains in the context of a biofilm. While the absence of antibiotic once again had no effect on the planktonic outgrowth of bacteria, treatment with ADEP-4 successfully eradicated biofilms of both strains (Fig. 4A and B). These data indicate that strains harboring the *relA* mutation that conferred enhanced tolerance to clinically approved antibiotics during biofilm growth remained susceptible to this investigational class of compounds.

## DISCUSSION

The stringent response is a highly conserved mechanism by which bacteria can control broad metabolic changes required for survival under adverse conditions and has been implicated in the virulence of several prominent pathogens (41). It has also been implicated in the ability to promote antibiotic “tolerance”: bacterial cells that are able to survive antibiotic concentrations much higher than the MIC and can replicate once antibiotic pressure is removed (15, 17, 19, 22, 24). It is hypothesized that this is due to the absence or inactivity of antibiotic targets: for example, tolerant persister cells may not be actively dividing, so the presence of linezolid would not affect protein synthesis and therefore would not cause bacterial cell death. As such, these tolerant cells can persist during prolonged antibiotic exposure, further complicating antibiotic treatment.

In *Enterococcus*, two enzymes are responsible for the production of the stringent response alarmone (p)ppGpp: the bifunctional RSH (i.e., Rel SpoT homolog [RelA for this study]) and the monofunctional RelQ alarmone synthase (42, 43). Recent studies have highlighted the importance of subtle alterations in the levels of basal ppGpp as opposed to the higher levels induced during the stringent response in mediating broad-range antibiotic tolerance (42). These data suggest that mutations in *relA* that alter basal ppGpp levels may confer antibiotic tolerance of significance in an immunocompromised setting.

**FIG 5:**
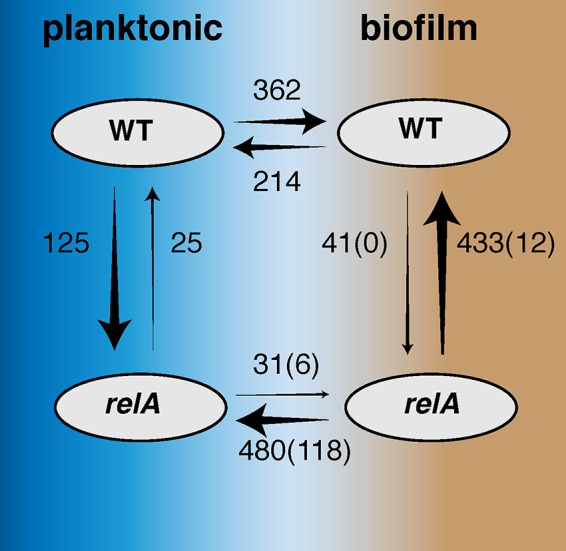
Summary of significant differences in expression between the WT and the *relA* mutant, in both planktonic and biofilm growth, as revealed by RNA-seq. The numbers shown are the number of genes in which expression differs at a very stringent cutoff of *q* < 0.001; arrow direction should be read as “is less than.” Arrow thickness is proportionate to the number of differences. Boxes containing both arrows denote genes with divergent expression, as follows: 35 genes have reduced expression in the *relA* mutant biofilm that were increased in the WT biofilm, and 25 genes have reduced expression in the *relA* biofilm that were increased in the *relA* planktonic culture.

Emergence of a mutation in the *relA* gene has been observed during persistent infection with *S. aureus*, but not in *Enterococcus* (22). Furthermore, the identification of a clinical VRE isolate with tolerance to multiple antibiotics due to a *relA* mutation has not been previously reported. It is important to note that our antibiotic-tolerant *relA* VRE mutants could not be distinguished from their isogenic WT counterparts by standard *in vitro* clinical MIC testing or by traditional growth curves (Fig. S1A) and time-kill assays (Fig. S2) of exponentially replicating cells in liquid culture. While the fitness advantage of the *relA* mutation was not present in planktonic culture, we determined that it was significant in the biofilm elimination model, whereby the mutant survived exposure to several antibiotics used to treat infection, suggesting an increase in antibiotic tolerance (Fig. 5). This has previously been reported for multiple bacterial pathogens, but not for VRE (22, 44). Biofilms harboring the RelA missense mutation in the hydrolase domain had increased resting alarmone levels and were recalcitrant to elimination by conventional antibiotic treatment. This included both linezolid and daptomycin, of which no genetically encoded antibiotic resistance mechanisms existed (Table 2). However, both the WT and *relA* mutant were effectively eradicated by ADEP-4, which has been shown to constitutively activate the housekeeping protease ClpP. These data indicate that such unconventional antibiotic strategies may be extremely effective for eliminating tolerant, persistent infections in an immunocompromised host. The ClpP-sensitive, conventional antibiotic-tolerant biofilm population is reminiscent of the presumed clinical course, as the focus of infection is thought to have initially been the CVC followed by possible endocarditis, both infections typically thought to be caused by multicellular biofilm communities. Despite antibiotic therapy, bacterial clearance required host immune reconstitution; the infection only cleared upon neutrophil recovery. This is an important reminder that clinical response may not correlate with *in vitro* susceptibility testing, especially in immunocompromised hosts or biofilm-associated infections (34).

**FIG 6:**
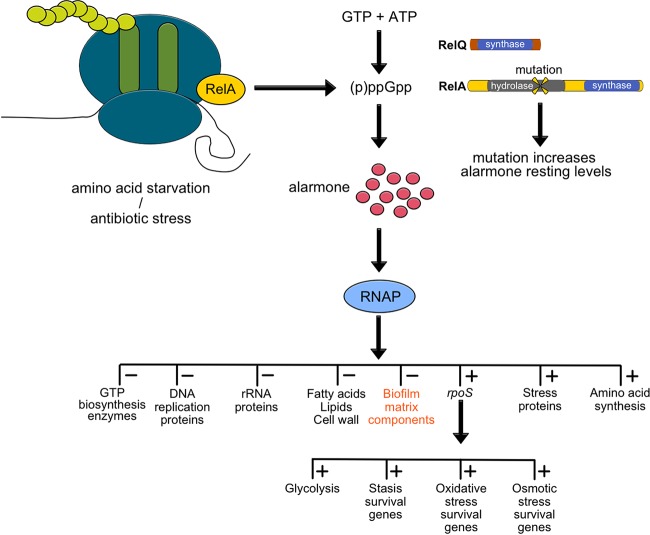
Pathway of stringent response with *relA* hydrolase mutation. During stress response, RelA (RSH/SpoT homolog in VRE) and RelQ are responsible for the production of the alarmone (red circles). In the *relA* mutants, a missense mutation adjacent to an active site in the hydrolysis domain led to increased resting levels of ppGpp. Cells were “primed” to respond faster to stress in a biofilm, which included antibiotic stress in the patient’s case. We propose that the presence of ppGpp downregulated multiple nonessential pathways, as shown by the RNA-seq data, including biofilm components. Therefore, while the *relA* mutant was fit to survive in an immunocompromised patient, once the immune system was reconstituted, the fitness trade-off of a smaller, less robust biofilm would enhance susceptibility to neutrophil killing.

There exist a multitude of pathways by which bacteria can evolve greater antibiotic tolerance, as evidenced by *in vitro* selection; however, only a subset of the mutations observed in the laboratory are identified in clinical isolates (45). Mutations conferring tolerance provide an obvious advantage to bacteria in the presence of the antibiotic but may involve trade-offs in fitness (46). In order for new tolerance mechanisms to disseminate throughout a population, any fitness trade-off must be alleviated through compensatory mutations to allow the strain to compete with and outgrow nonresistant organisms in the absence of antibiotic selection. In immunocompromised hosts, bacterial pathogens may replicate and evolve novel antibiotic tolerance mechanisms free from the usual immune surveillance (47–49). This phenomenon is reflected in our study, whereby a mutation in the stringent response arose and was maintained under antibiotic pressure in the context of an ongoing, profoundly immunocompromised state (Fig. 6).

While *in vitro* data suggested that the presence of the *relA* mutation rendered these strains more tolerant to antibiotic stress within a biofilm, we detected a fitness defect in the ability to form biofilms of the same size compared to the isogenic WT strain. In accordance with patient treatment (Table 1), while the immune system was suppressed, the *relA* mutants were more adapted to tolerate antibiotic stress during the multiantibiotic treatment phase, which we hypothesized was due to higher resting alarmone levels. Although they were able to persist within the bloodstream of the immunocompromised patient, these *relA* mutants were deficient in producing a larger biofilm, as shown in confocal microscopy results. It is well known that RelA activation and alarmone production are responsible for the downregulation of nonessential genes and proteins, including proteins and saccharides that form biofilm matrices (50).

As discussed, bacteremia finally resolved only upon neutrophil recovery. These findings indicate a “perfect storm” for antibiotic tolerance development: the compromised host immune system ameliorates the impact of any bacterial fitness loss and provides a permissive environment for replication coupled with continuous antibiotic selection pressure due to the empirical therapy required for treatment or prophylaxis. This specific set of requirements could be relevant in several other immunocompromised patient populations in which rates of bacterial colonization and infection are high, especially if they are biofilm mediated.

This study has some limitations. Although persistence of bacteremia clinically suggested an intravascular focus such as endocarditis, this was never proven. Similarly, the coexistence of both the mutant and parent strains throughout the course of infection might represent a mixed biofilm at one site or infection with different isolates at discrete anatomic locations. Population profiling might have improved the interpretation of these results, but only a single sample was saved by the clinical microbiology laboratory at each time point, making such profiling impossible to perform. Generation of a *relA* missense mutation in the WT VRE background could provide further weight to the conclusions of the study, but genetic manipulation of a multidrug-resistant enterococcal strain has the potential to introduce additional mutations arising during prolonged passaging. Hence we opted to compare strains that were isogenic, with the exception of the *relA* SNP, based on genome sequencing for all phenotypic comparisons.

Based on our results, we conclude that during prolonged infection, bacterial populations may acquire evolutionarily advantageous mutations conferring antibiotic tolerance. This may be especially likely within the permissive environment of an immunocompromised host. This is the first clinical case showing *in vivo* development of a mutation in the enterococcal *relA* gene during prolonged infection that functionally conferred tolerance to clinically relevant antibiotics without a change in clinically tested MIC. The case expands our understanding of the role of the stringent response in susceptibility and tolerance to a wide range of antibiotics, especially in biofilms, and demonstrates that these mutations can occur during human infection. This mutation is especially clinically significant, as linezolid and daptomycin are the last line of defense against infection with VRE, an important and already highly resistant pathogen. Furthermore, ClpP activators retained bactericidal activity against the *relA* mutant within a biofilm, indicating a potential future therapeutic strategy for targeting such persistent infections.

## MATERIALS AND METHODS

### Initial growth and storage of VRE isolates.

Twenty-two VRE bloodstream isolates, one from each day of collection, were received from the clinical microbiology laboratory at St. Jude Children’s Research Hospital. A loop of each strain was grown in Todd-Hewitt broth supplemented with 3% yeast extract (ThyB [Bacto BD]) at 37°C in 5% CO_2_. Glycerol stocks were generated for each strain and stored at −80°C until further analysis. Each glycerol stock was also grown on tryptic soy agar (21) containing 3% sheep blood (Merck, Darmstadt, Germany) to verify purity: colony morphology results indicated that all samples were pure.

### Genomic DNA extraction and sequencing.

Bacteria were grown in unshaken overnight cultures in ThyB medium and collected by centrifugation before genomic DNA was extracted by using the Purelink Genomic Extraction kit (Life Technologies, Inc.). Sequencing libraries were prepared and bar-coded by using the Nextera kit (Illumina) and pooled in one lane of an Illumina HiSeq2500, yielding a mean (standard deviation [SD]) of 6.6 × 10^6^ (4.2 × 10^6^) reads and 1.0 Gbp ± 637 Mbp. To obtain a more robust genome assembly, the initial bloodstream isolate was also subjected to PacBio sequencing with 5- to 10-kb fragment libraries loaded on one SMRTcell (Johns Hopkins Sequencing Center), producing 385.2 Mbp of sequence. Contiguous consensus sequences (contigs) were assembled by using the PacBio SMRTanalysis toolkit, resulting in a genome containing 3,242,672 Mbp in four circular contigs and 118.8× mean per-bp coverage. This polished genome served as a reference for the mapping of all subsequent Illumina short reads, at a mean per-base-pair coverage of 256.6× (SD, 138×). Reference mapping and the detection of SNPs, indels, and structural variants were conducted by using breseq software as described previously (51). Mutation calls were pooled by using a custom shell script, 23 of which were used to correct the PacBio genome assembly at sites that were mostly restricted to one plasmid. A preliminary set of nine putative unique calls were verified by manually inspecting the sequence pileups, which revealed 4 false mutations produced by poor alignments and 5 consensus mutations among the 22 bloodstream isolates, including one in the *relA* gene responsible for the stringent response.

### *relA* sequencing.

A single VRE isolate from each of infection days 1, 3, and 5 (wild type) and 4, 16, and 17 (mutant) was grown in 5 ml ThyB overnight at 37°C. Cultures were bead beaten with silica beads for 10 min to break open cells. An internal fragment of the* relA* gene of each isolate was PCR amplified by using forward primer 5′-GTGGACGGCGTAACCAAATTAGGG-3′ and reverse primer 5′-CCACTTACGTATTTTTCACGTTCTTCTC-3′. DreamTaq Green DNA polymerase (Thermo Scientific) was used with a 51°C annealing temperature and a 1-min extension period at 72°C for 30 cycles. PCRs were cleaned up by using Qiagen MinElute kits and sequenced by using the forward primer with Sanger sequencing to confirm the *relA* SNP.

### Planktonic growth measurements.

Ninety-six-well plates (Costar) were inoculated with approximately 2 × 10^6^ CFU/ml of either the *relA* WT or isogenic *relA* mutant per well. Two hundred microliters ThyB was used to measure the growth of each strain in the presence of linezolid (6.25 µg/ml) over 8 h. After 8 h, triplicates of each strain in either no drug or linezolid were serially diluted on ThyB agar and plates were incubated at 37°C overnight. The number of CFU per milliliter was calculated, and growth of each strain was compared to growth at 8 h in no drug. Two-tailed *t* test was used to calculate significant differences in the growth of each strain, of which there were no significant differences observed.

### Time-kill kinetics.

VRE isolates corresponding to the WT (day 5) and *relA* mutant (day 17) were grown overnight in ThyB (pH 6.5) at 37°C. Cultures were back-diluted 1:100 or 1:1,000 to produce an approximate starting culture of 10^5^ CFU/ml. To obtain the daptomycin kill kinetics, sterile-filtered (0.22-µm-pore filter) 1 mM CaCl_2_ was added to ThyB prior to the experiment. Aliquots (1 ml) of each culture were separately analyzed over time (in triplicate), and either dimethyl sulfoxide (DMSO)/PBS (no-compound control [Sigma/Lonza]), 50 µg/ml daptomycin (Cubist Pharmaceuticals), or 200 µg/ml linezolid (Sigma) was added. Each sample was analyzed by performing serial dilution to determine the number of CFU per milliliter over time. The limit of detection was 1 × 10^3^ CFU/ml. Each time-kill assay was repeated three times, and a representative replicate is presented (see Fig. S2 in the supplemental material).

### Biofilm antibiotic tolerance assays.

Nunc plates with peg lids were used to grow VRE biofilms and analyze the biofilm eradication capabilities of linezolid, vancomycin, and daptomycin. A 200-µl aliquot of ThyB (+1 mM CaCl_2_ for daptomycin) was inoculated into single wells of the 96-well plates. Each condition was tested at least in triplicate for WT and the isogenic *relA* mutant. Frozen stocks of VRE from days 5 (22) and 17 (*relA* mutant) were inoculated into each well. Peg lids were seeded into the liquid culture, and the cells were allowed to grow overnight at 37°C. Peg lids were then washed in sterile water to remove planktonic cells, placed in new Nunc plates containing ThyB and under the appropriate drug/stress condition, and incubated at 37°C overnight. A second wash in sterile water was then performed, and the peg lid was placed into a new Nunc plate with 100 µl ThyB. Centrifugation at 800 × *g* for 20 min was performed to remove biofilms from the peg. After centrifugation, peg lids were discarded, and the plate was incubated at 37°C overnight in a Cytation 3 (BioTek) plate reader recording absorbance at 600 nm over 24 h. This method allowed quantification of the growth of any viable cells from the biofilms. The average of at least three independent readings is reported for each strain and condition.

### Stringent response measurement.

Strains were grown at 37°C in C+Y medium (53) without potassium phosphate until an optical density at 600 nm (OD_600_) measurement of 0.2 was obtained. To a 1-ml aliquot of culture, 500 μCi of [^32^P]orthophosphate (American Radiolabeled Chemicals) was added. After a 2.5-h incubation, a 100-μl aliquot of culture was added to 50 μl of 13 M formic acid and then exposed to two freeze/thaw cycles using dry ice. Samples were spotted onto a polyethyleneimine (PEI)-cellulose TLC plate (Analtech) and developed in a tank of 1.5 M KH_2_PO_4_ (pH 3.4). After the plate was dried, it was exposed to a phospho-imaging screen for 48 h; the signal intensity was read by using a Typhoon FLA 9500 (GE Healthcare Life Sciences) and quantified by using ImageQuant TL. Five replicates harboring a WT *relA* locus and five replicates harboring the point mutation of interest were included in these experiments. As a control, each VRE strain was incubated with 100 µg/ml mupirocin (Sigma) as a positive control for stringent response induction. In addition, strains were also incubated with 10 µg/ml linezolid to determine whether linezolid exposure induced the stringent response.

### Biofilm quantification assays.

Twenty-four-well plates (Costar) were coated with bovine plasma fibronectin (Sigma) by an overnight incubation at 37°C. Each well was gently washed with 1 ml of 1× PBS, followed by addition of 1 ml fresh ThyB and inoculated from frozen titered stocks of the VRE WT or *relA* mutant. Biofilms were allowed to form by overnight growth without shaking at 37°C. Supernatant was removed, and each biofilm was gently washed twice with 1× PBS to remove planktonic bacteria. PBS was discarded, and wells were allowed to dry. Four hundred microliters of 1% (wt/vol) crystal violet was added to each well or 1 µl PBS to disrupt biofilms for determination of CFU per milliliter. After 20 min for staining, crystal violet was removed, and each well was washed multiple times with 1 × PBS until supernatant was clear. After drying, 1 ml 95% ethanol was added to each well, and the well was gently shaken for 10 min to allow complete solubilization of the crystal violet into solution. Absorbance at 595 nm was measured using a Cytation3 plate reader (BioTek) to quantify biofilm. At least three independent determinations for each experimental condition were performed. The number of CFU per milliliter for each biofilm was determined via serial dilution and plating on ThyB agar.

### Fluorescence microscopy of VRE biofilms.

μ-slide 8-chamber microscopy slides (IBIDI) were inoculated (each chamber) with 300 µl ThyB per well plus 3-µl frozen stocks of the VRE *relA* WT or mutant. Slides were incubated overnight at 37°C to allow biofilms to form. Two hundred microliters of medium was carefully removed from each chamber in order to not disrupt formed biofilms, 200 µl fresh ThyB was added, and slides were again incubated overnight at 37°C. One hour prior to confocal microscopy, components A and B of the Thermo Scientific Live/Dead BacLight bacterial viability kit were thawed and equally mixed according to the manufacturer’s instructions. One microliter of this mixture was then carefully added to wells at a staggered interval of 30 min, to allow comparable microscopy pictures of live/dead staining to occur. After a 30-min incubation at room temperature in the dark, the first chamber was analyzed in a confocal microscope (Nikon C2). For each chamber, four random segments were subject to z-stacking at a total height of 16 µm from the first fluorescent image corresponding to the bottom of the biofilm. Total green (fluorescein isothiocyanate [FITC]) and red (tetramethyl rhodamine isothiocyanate [TRITC]) filter sets from this volume (16 µm^3^) were analyzed for each segment, and each strain was analyzed in at least 2 separate chambers. This allowed an *n* value of 8, corresponding to four individual segments per chamber. The ratio of green to red signal was calculated from the average of each section, and results were combined for the final average value reported. Prism6 was used to analyze all data, and significance was calculated via two-tailed *t* test.

### RNA extraction, cDNA synthesis and qRT-PCR, rRNA depletion, and RNA sequencing.

Seventy-five-square-centimeter flasks (Corning) were coated overnight at 37°C with fibronectin (Sigma). After 24 h, each flask was inoculated with 30 ml ThyB and either the *relA* WT or mutant. After 24 h, supernatant was removed, and biofilm cells were scraped off the flask, eluted in 10 ml RNAprotect, and pelleted by a 10-min spin at 10,000 × *g*. Planktonic cells were grown in 30 ml ThyB until mid-log phase (OD_600_ of 0.5), suspended in RNAprotect, and pelleted by a 10-min spin at 10,000 × *g*. Each biofilm sample was comprised of 3 flasks that were subsequently extracted and pooled to generate sufficient RNA for sequencing. All samples were run in triplicate for planktonic or biofilm pellets for each strain, and RNA extraction was performed immediately after collection of the cell pellets.

RNA extraction was performed using the Qiagen RNeasy kit with slight modifications for initial cell lysis. Briefly, 250 µl silicon beads was added to each pellet containing RLT lysis buffer and 2-mercaptoethanol and bead beat (FastPrep MP Biomedicals) for one cycle of 45 s with a setting of 5.5. After cell lysis, the Qiagen RNeasy process was continued with a QiaShredder cleanup step and the RNeasy protocol was completed. Each RNA sample was eluted in a final volume of 30 µl of RNase-free water and stored at −20°C. rRNA was depleted using the RiboMinus kit (Thermo-Fisher) following the manufacturer’s guidelines. For each sample, 30 µg of RNA was utilized for the depletions. Following depletion, libraries were prepared for sequencing using the Illumina stranded mRNA protocol.

### RNA-seq analysis.

Four replicates each of the *relA* WT and mutant strains were grown under planktonic and biofilm conditions, and RNA was extracted from bulk cultures. RNA libraries were prepared for sequencing and pooled on one lane of an Illumina HiSeq2000 at St. Jude Children’s Research Hospital. These produced an average of 82 million reads for the WT planktonic cultures, 91 million reads for the WT biofilm cultures, 82 million reads for the *relA* planktonic cultures, and 130 million reads for the *relA* biofilm cultures. Differences in expression were evaluated using Rockhopper version 2.03 (52), which compares strand-aware, normalized sequence counts found throughout the reference genome. We used the well-annotated *E. faecalis* Aus04 reference genome (PRJNA86649) for this purpose, which had high identity to the strains reported here. Resulting *P* values of differentially expressed genes were used to compute false discovery rate (*q*) values derived from a Benjamini-Hochberg correction with a false discovery rate of <1%. Only *q* values of <0.001 are reported here in Table S2 in the supplemental material, almost certainly underestimating the number of significant differences.

10.1128/mBio.02124-16.4 Table S2  RNA-seq expression analysis. Download Table S2, XLSX file, 1.4 MB. Copyright © 2017 Honsa et al. 2017 Honsa et al. This content is distributed under the terms of the Creative Commons Attribution 4.0 International license.

### Ethics statement.

This project was approved by the St. Jude Children’s Research Hospital Institutional Review Board before any research procedures were performed (XPD14-052). Consent was obtained in writing from the patient’s legal guardian for use of clinical samples and access to the medical record for research purposes.

### Accession number(s).

The chromosomal and plasmid sequences of the initial isolate are accessible at NCBI under GenBank accession no. CP018070, CP018071, CP018072, and CP018073.
