# Supplementary material for: RelA Mutant Enterococcus faecium with Multiantibiotic Tolerance Arising in an Immunocompromised Host
Source: mBio. 2017 Jan 3;8(1):e02124-16. doi: 10.1128/mBio.02124-16 (PMC5210501; doi:10.1128/mBio.02124-16)
Supplement: Figure S1 [file mbo006163120sf1.docx]

**Supplemental Figure 1. No planktonic growth defects were detected in either WT or isogenic *relA* mutant.** A. Each strain [*relA* WT (black) or isogenic mutant (red) [35]] was inoculated in a 96-well plate with 200 µL ThyB per well, without any stressors. Growth was monitored by measuring absorbance at 600 nm at 37 ^o^C in a Cytation3 plate reader. Error bars represent mean standard deviation, and represents at least 6 independent analyses. B. Comparable starting inoculums of *relA* WT (black) or mutant [35] were inoculated into 200 µL ThyB in a 96-well plate. 6.25 µg/mL linezolid was added to triplicate samples, and growth at 8-hours post treatment was compared to no drug controls by serial dilution plating for CFU/mL detection. No significance was detected; error bars represent average deviation.
