# Supplementary material for: RelA Mutant Enterococcus faecium with Multiantibiotic Tolerance Arising in an Immunocompromised Host
Source: mBio. 2017 Jan 3;8(1):e02124-16. doi: 10.1128/mBio.02124-16 (PMC5210501; doi:10.1128/mBio.02124-16)
Supplement: Figure S2 [file mbo006163120sf2.docx]

**Supplemental Figure 2. Time kill assays of WT and *relA* mutant.** VRE isolates corresponding to WT and *relA* mutant were grown overnight in ThyB pH 6.5 at 37°C. Cultures were back-diluted to produce a starting culture of 10^5^ CFU/mL. A) 50 µg/mL daptomycin and 1 mM CaCl_2_, or B) 200 µg/mL linezolid was added. Each sample was analyzed by performing serial dilution to determine the CFU/mL over time. The limit of detection was 1 × 10^3^ CFU/mL. Each time kill assay was repeated three times and a representative figure is presented.
